# Study of Effect of Impacting Direction on Abrasive Nanometric Cutting Process with Molecular Dynamics

**DOI:** 10.1186/s11671-017-2412-2

**Published:** 2018-01-11

**Authors:** Junye Li, Wenqing Meng, Kun Dong, Xinming Zhang, Weihong Zhao

**Affiliations:** College of Mechanical and Electric Engineering, Changchun University of Science and Technology, Changchun, 130022 China

**Keywords:** Molecular dynamics, Nanometric cutting, Impacting direction, Monocrystalline copper

## Abstract

Abrasive flow polishing plays an important part in modern ultra-precision machining. Ultrafine particles suspended in the medium of abrasive flow removes the material in nanoscale. In this paper, three-dimensional molecular dynamics (MD) simulations are performed to investigate the effect of impacting direction on abrasive cutting process during abrasive flow polishing. The molecular dynamics simulation software Lammps was used to simulate the cutting of single crystal copper with SiC abrasive grains at different cutting angles (0^o^–45^o^). At a constant friction coefficient, we found a direct relation between cutting angle and cutting force, which ultimately increases the number of dislocation during abrasive flow machining. Our theoretical study reveal that a small cutting angle is beneficial for improving surface quality and reducing internal defects in the workpiece. However, there is no obvious relationship between cutting angle and friction coefficient.

## Background

**Fig. 1:**
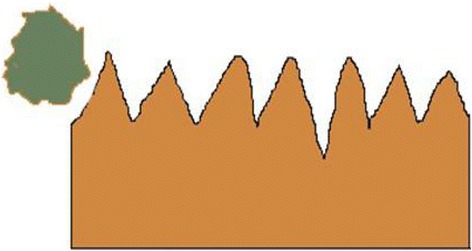
Sketch of abrasive cutting workpiece. All of the figures are about the effect of impacting direction on abrasive nanometric cutting process with molecular dynamics. Figure 1 is the sketch of abrasive cutting workpiece. In the abrasive flow polishing process, the suspended particles in the medium will flow along the media at a certain speed with the impact of micro cutting workpiece surface

In modern ultra-precision machining, material removal technologies play an important role in microelectronics, micromechanical, and optical element manufacturing. The demand of miniaturized devices with high dimensional accuracy and quality surface, making ultra-precision processes the major choice in the mentioned field [1]. Moreover, the changes of surface components and sub-surface structure are at the nanometer length scale. Abrasive particle flow polishing technology plays an important role in many fields of precision machining and is just like other non-traditional finishing technologies which improve surface quality. This technology has attracted lots of researchers due to its significant role. E. Uhlmann and other researchers have reported the computer simulation of abrasive grain polishing ceramic surface for the designed experiments to verify the grinding fluid flow of various processing factors on the effect of cutting materials [2]. Sehijpal Singh and other researchers use the abrasive flow polishing technology for cutting copper and aluminum materials. From scanning electron microscopic analysis, they found a deep groove surface of their workpiece [3]. G. Venkatesh and other researchers have reported ultrasonic assisted abrasive grain polishing technology for the conical gear on the complex surface of finishing process. In this technique, the abrasive grain velocity is higher than the conventional abrasive grain flow to collide with the surface of workpiece, which can improve the processing efficiency. From their experimental and theoretical methods, they found that this technology is one of the best choices for gear blade finishing [4, 5]. K. Kamal et al. studied the rheological properties of the abrasive liquid in the fluid abrasive viscosity, shear rate, and creep time [6]. However, most of the abrasive flow polishing studies are based on macro level and very rare attention has been paid to micro level. In the abrasive flow polishing process, the suspended particles in the medium will flow along the media, at a certain speed with the impact of micro cutting workpiece surface (Fig. 1).

As the shape of the abrasive grains is not regular, having certain edges and corners which act on the surface of the workpiece, similar to the tool. But the cutting process is on the atomic scale which is obviously different from the material removal process. A nanoscale cutting involves few nanometers or less of the material surface, but it is very difficult to observe this process by experiments. Therefore, MD simulation as a theoretical investigation method is very useful in studying the nanometric cutting process. Molecular dynamics as a computer simulation technique, which uses a time-based statistical mechanics method to study the interrelation of atoms for conditions prediction and analysis. This is also a powerful tool for simulating and understanding materials removal processes. In the literature, there exist numerous studies regarding MD as a tool to investigate precision machining. Oluwajobi and Chen have done extensive work on MD simulation of nanoscale machining of copper [7]. In their studies, they investigated various parameters for nanomachining such as minimum cut depth, geometry, and interatomic potential [8]. In addition, MD simulations results have also been successful in the past to address number of problems concerning the nanometric cutting process of brittle materials such as silicon [9]. Komanduri et al. conducted an MD simulation for nanometric cutting of single-crystal of pure silicon defect-free, using the Tersoff potential. They studied the effects of rake angle, width of cut, depth of cut, and clearance angle on material removal and surface generation [10]. Goel et al. investigated the atomistic aspects of ductile response of SiC during the nanometric cutting process. They discovered the presence of a sp3-sp2 order-disorder transition which finally resulted in the graphitization of diamond [11]. Cai et al. used MD to study the nanoscale ductile mode cutting of silicon. Their reported the tool cutting edge and its effects on the shear stress in the workpiece material [12]. Arafin et al. has discussed the effect of cutting edge radius in nanoscale ductile mode cutting of silicon wafer [13]. Various other researchers have also highlighted the different conditions in nanomachining of silicon using MD simulations. There investigations consist of cutting forces, depth of cut, temperature, shear stress, and other parameters. However, there is a lack of adequate experimental validations in this area. Qihong Fang et al. studied the interaction mechanism of dislocations in different heterogeneous materials with different scales, and the dislocation and the relationship between material toughness and fracture damage. They use molecular dynamic simulations to study the nanoindentation onto three different crystal structures including the single crystalline, polycrystalline, and nanotwinned polycrystalline copper. With the increase of scratching rate, scratching force and workpiece temperature increase continuously due to severe plastic deformation and large chip volume, resulting in dislocation slip, GB slip, and twinning/detwinning [14, 15].

### Methods/Experimental

**Fig. 2:**
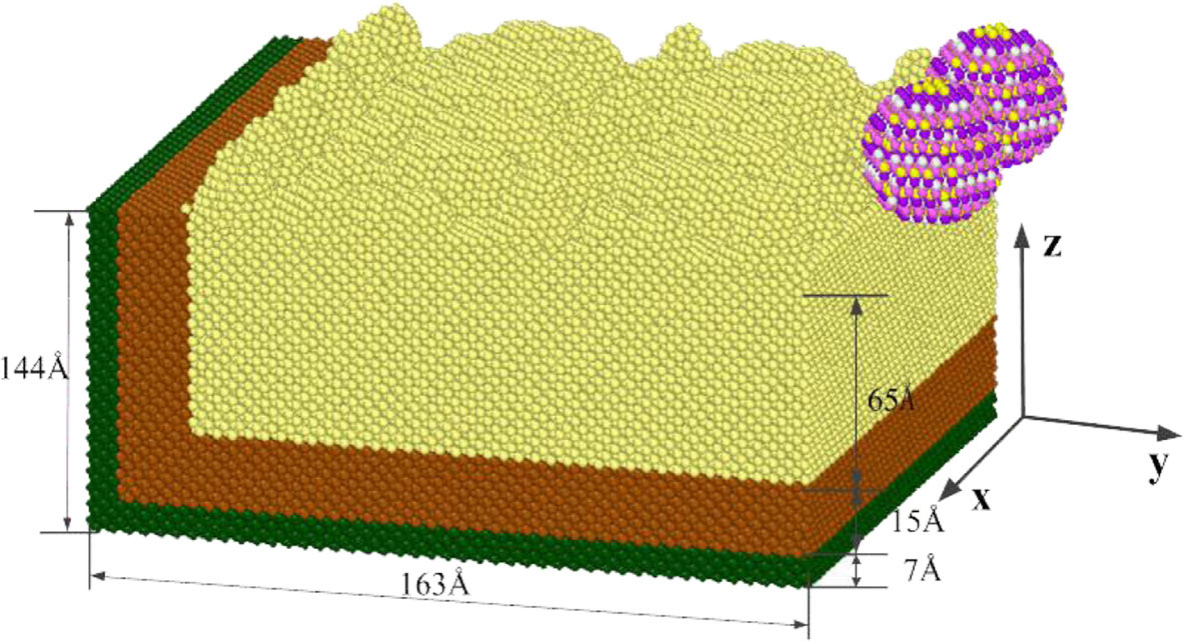
Simulation model of SiC abrasive cutting single crystal copper. Figure 2 is about the simulation model of SiC abrasive cutting single crystal copper; we built two SiC abrasive for cutting single crystal copper. In the abrasive flow polishing experiment, the workpiece was usually cut with SiC abrasive. After the construction of SiC abrasive model, the molecular dynamics simulation of the workpiece was carried out. The model of two SiC abrasive cutting monocrystal copper with different angles was constructed

In the abrasive flow polishing experiment, the workpiece was usually cut with SiC abrasive. After the model of SiC abrasive grains was established, the molecular dynamics simulation of the workpiece was carried out by the software Lammps. The model of two SiC abrasive cutting monocrystal copper with different angles was constructed. A simulation model of a SiC abrasive grains crash cutting monocrystal copper is shown in Fig. 2.

The size of the model is shown in Fig. 2 while the number of simulated atoms was 159,020. The abrasive radius is 15 Å, total number of C atoms and Si atoms in the abrasive grains are 1406. And the numbers of carbon and silicon atoms were 681 and 725, respectively. The relaxation step number is set to 10,000 steps for the simulated equilibrium ensemble, collision simulation steps to 100,000 cutting step, each step of the simulation was 0.001 ps, cutting simulation, the SiC abrasive particles in the cutting direction speed of 80 m/s. AFM analysis value grains collide micromachining workpiece during polishing, SiC abrasive grains to explore the molecular collision dynamics different angles cutting process. In the process of abrasive flow cutting, the cutting force increases as the cutting speed increases, and the size of the cutting force directly affects the cutting effect. Therefore, the choice of reasonable cutting speed has an important impact on the quality of cutting. To achieve a high-quality cutting, we chose the cutting speed of 80 m/s, because it produces the cutting force which can quickly destroy the interaction between the workpiece atoms [16].

**Fig. 3:**
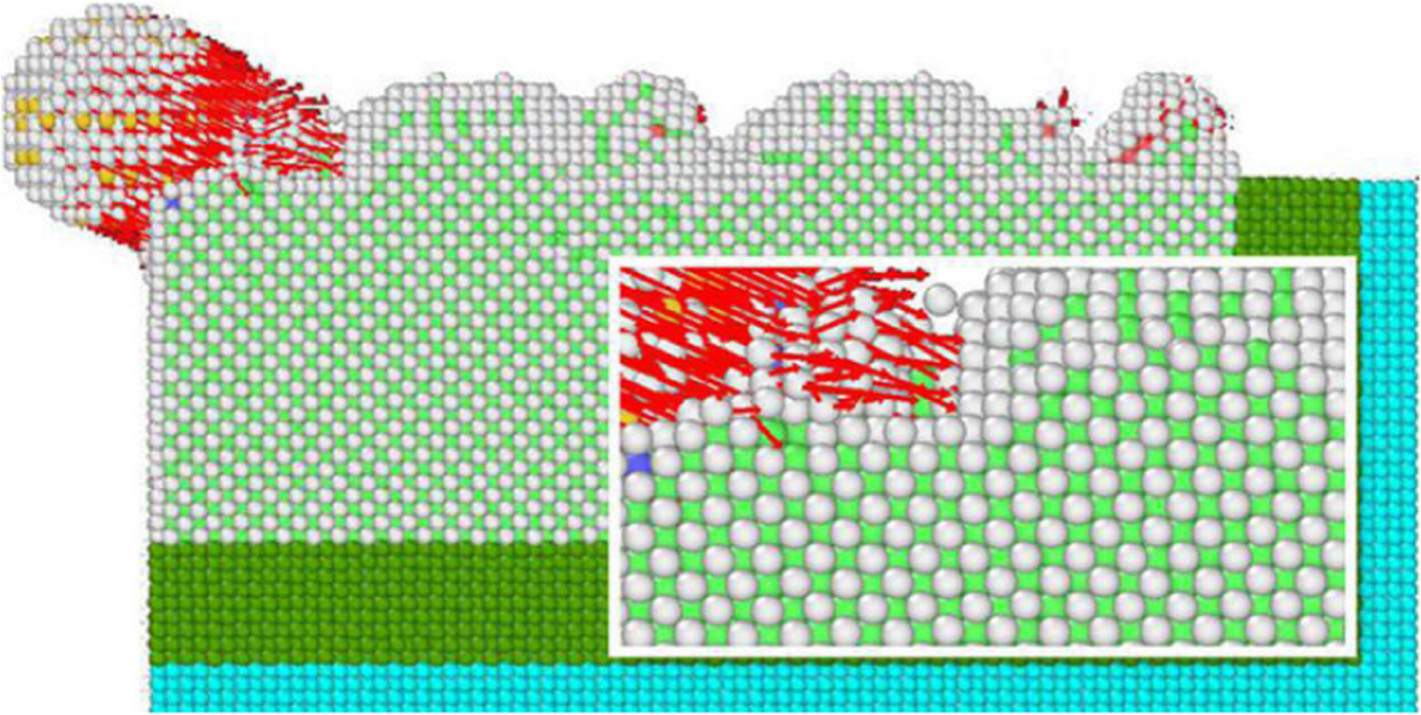
Low velocity atomic displacement

**Fig. 4:**
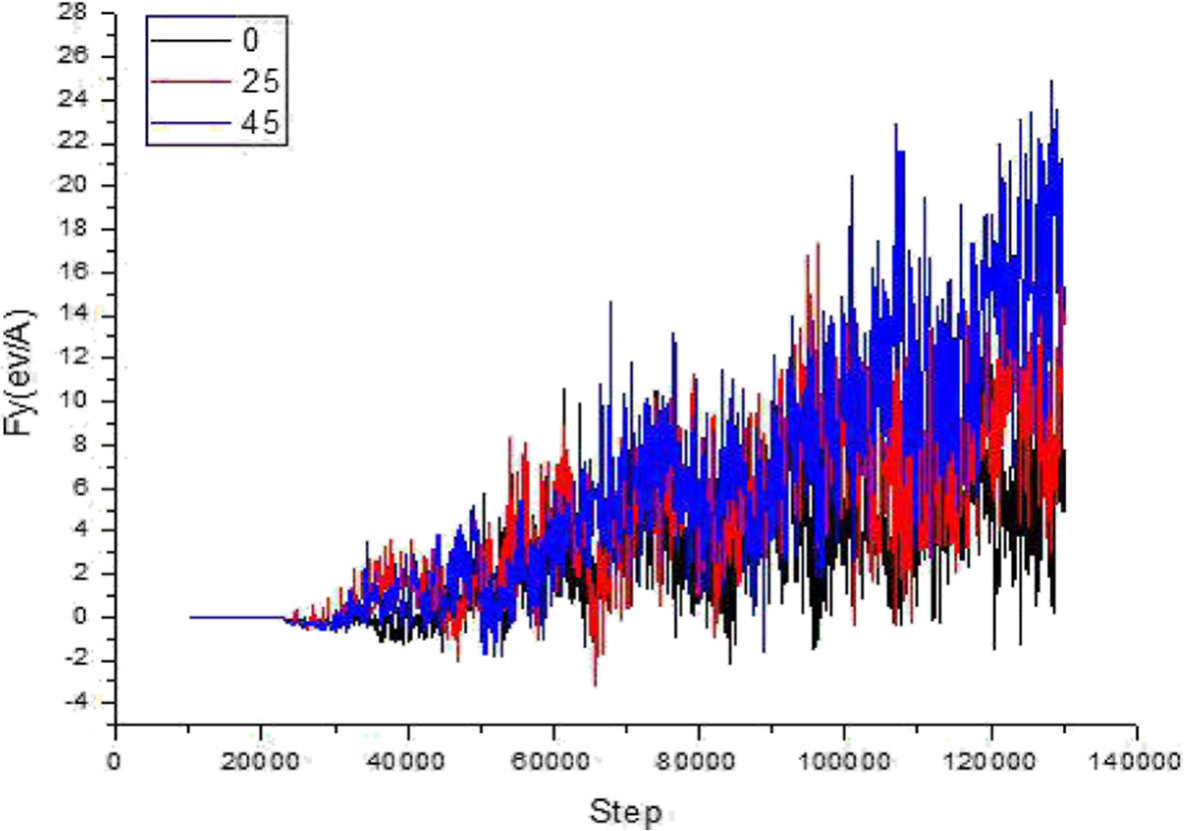
The relationship between the cutting force, the cutting angle, and the simulation step

In molecular dynamics simulations at low cutting speed such as 10 m/s, atomic displacement maps and the pattern how cutting force of silicon carbide abrasive particles and cutting angles vary with simulation step size, as shown in Figs. 3 and 4. Because the cutting speed is too small, the cutting force is also inadequate, resulting in far less deformation and dislocation of the lattice. On the whole, the cutting efficiency is low and the cutting quality is relative poor. By contrast, when the cutting speed is up to 80 m/s in microscopical experiment, chemical bonds between copper atoms can be broken quickly and effectively, so as to achieve the goal of instant high quality cutting. Therefore, after a comprehensive analysis, 80 m/s cutting speed is reasonable for the simulation.

In order to simulate the feasibility and credibility, we choose a reasonable empirical potential function to consider the interatomic potentials of atoms. According to the different objects, we can divide the potential function into pair potential function and many-body potential function. The two models between single crystal copper and abrasive grains describe the interatomic potentials of atoms between them by Morse potential [17], the EAM potential is used to describe the interatomic potentials of atoms between copper atoms [18–20], and the interaction between SiC particles is described using the Tersoff potential [21].

**Fig. 5:**
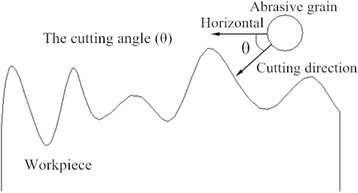
The sketch of cutting angle. Figure 5 is the sketch of the different cutting angles when the abrasive grains are cut. Abrasive grain from different directions cutting the workpiece material, the material will have the performance and processing quality will be different

During the polishing process, the collision direction of the abrasive grains in the medium is random; their trajectories are not exactly along the surface of the workpiece. During the micro-cutting process, the cutting direction of the abrasive grains is not always orthogonal to the workpiece material. The cutting angle refers to the degree of the angle between the cutting direction of the abrasive grain and the horizontal plane. It is positive when particles approach towards the workpiece surface. The sketch of the cutting angle is shown in Fig. 5.

In this study, MD simulation is employed to simulate multi-abrasives with different impacting direction of the cut monocrystalline copper. As shown in Fig. 5, the cutting angle is between 0° and 45° while the surface of copper workpiece is rough.

## Results and Discussion

### Analysis of SiC Abrasive Cutting Mechanics Collision

The impact of silicon carbide abrasive on the cutting of monocrystal copper material is achieved by destroying the lattice structure of single crystal of copper material. During the breakdown, interactions among copper atoms, the shearing stress imposed by C, Si atoms of SiC abrasive particles on the Cu atoms of workpiece material was identified to be the cutting force, which is an important physical parameter for the fact that the cutting force reflects the removal process of single crystal copper workpiece material profoundly. As discussed earlier, there is a big difference between micro and macro cutting force. Generally, macro-cutting force is the sum of cutting and grinding force, while in micro-cutting, the cutting force is generated by the interactions between the abrasive grains and the atoms of workpiece.

**Fig. 6:**
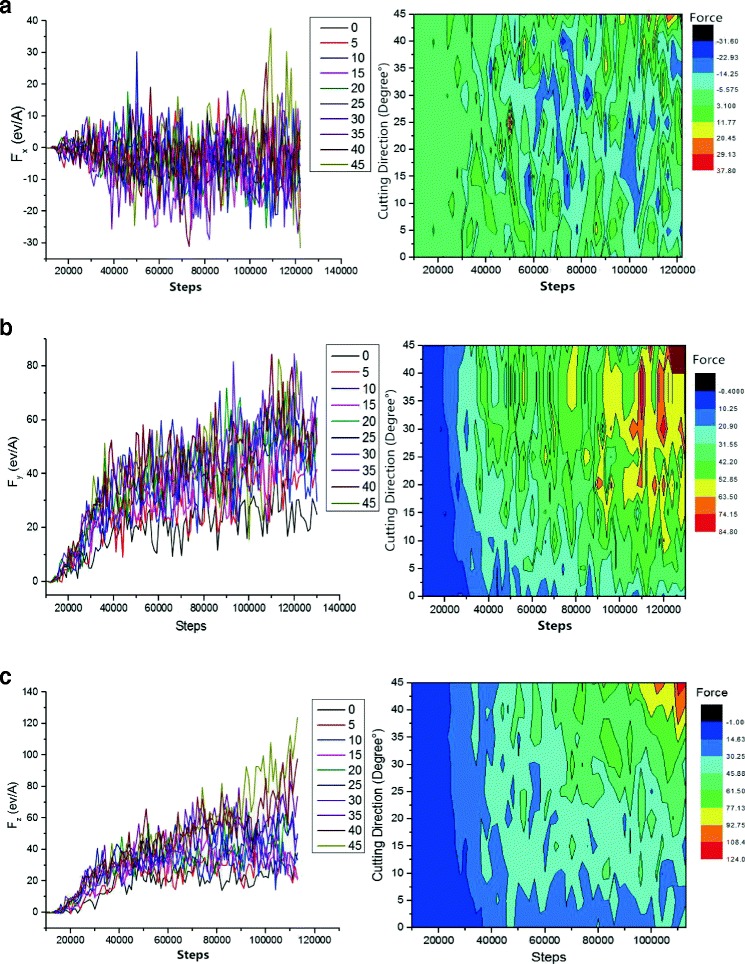
How cutting angle and simulation step size affect the cutting force. Figure 6 shows SiC abrasive cutting force and the cutting angle and simulation step diagram, with increasing cutting depth, the number of the crystal structure of destruction increases, the maximum value of the fluctuation of cutting force also becomes larger. **a** [100] direction of the cutting force. **b** [010] direction of the cutting force. **c** [001] direction of the cutting force

In Fig. 6, we have shown the changes of shear stress of abrasive grains in various directions, effect of shear stress with different directions, and the distribution of cutting forces along different cutting angles.

The silicon carbide abrasive grains cut and impacted on single crystal copper material in different angles edge such as [010] and [100]. The cutting forces along [001] and [010] directions were shearing forces. On further simulation, the cutting depth gradually increased which has persistently raised the cutting forces along direction [001] and [010]. However, up to a certain limit, these forces fluctuated as can be seen from Fig. 6. When abrasive particles moved towards the materials of workpiece, it has totally changed the interaction (from attraction to repulsion) between the outermost copper atoms and the atoms of SiC (Si and C). Initially, while grinding the workpiece material, cutting forces were inadequate due to the presence of repulsive forces of copper atoms. For the finishing process, the cutting forces should be sufficiently high so that the abrasive grains can easily break the chemical bonds in copper atoms to move freely. Furthermore, the cutting depth increment has direct relations with the atomic interactions. When the atoms began to pile up, the cutting force consistently increases until the abrasive particles enter workpiece, which required a steady state cutting force. In this relatively stable stage, the cutting-force fluctuation was subjected to the degree of lattice deformation, lattice reconstruction, amorphous phase change, and generation of chips. As the abrasive grains marched along the cutting direction, an external force was supplied upon the copper atom in the FCC lattice, which resulted displacements of the copper atoms. This displacement collapse the FCC lattice and transform it into a new lattice structures having dislocation. A similar situation is observed in the cutting force as well. There is no positive linear correlation between the cutting force along the direction of [010] and the collision angle of particles. In the collision with 0°, 5°, 10°, and beyond, the force in [010] direction was smaller and less fluctuated than those in other cutting directions. Cutting at smaller angle, basically remove burrs since the burr can be defined in height range of 3.5 ~ 15 Å. On comparing these directions with one another, if cutting depth is relatively smaller, the damage and deformation of crystal structure will be moderate. Therefore, in the whole cutting process, the shear force along the [010] direction was kept smaller, as shown in the curve graph of Fig. 6b. In the later stage of simulation, the cutting force reached to the minimum of 0°. It is also found that the cutting force has direct relation with angle as shown in Fig. 6c, which can be attributed to the fact that when particle velocity reached to 80 m/s, a larger cutting angle is achieved. On the other hand, at the same simulation step, the larger the component speed along [001] direction, higher angle, deeper particles cut, and the more atomic lattices were destroyed. However, there was a positive correlation between the cutting angle and cutting force. When grains moved along the [100] direction, frictions among C, Si, and Cu atoms were the source of cutting force. So, the cutting force did not show a gradual increasing trend. On further simulation, however, the cutting force increased and fluctuated, which was closely related to the deformation and reconstruction of the lattice as well as the generation of amorphous structural phase transition. Before the abrasive particles could destroy, the interaction between the atoms of the workpiece is achieved to remove material, which has ultimately increased the cutting force consistently. Upon increasing the abrasive cutting force, beyond the critical value (atom binding force), the atomic lattice damaged and the bonds dissociate which result an amorphous structure. On achieving this, the cutting force dropped to a relatively low value. The cutting force fluctuations appeared continuously during the whole process of abrasive flow machining, which is due to the increase of cutting depth, and more crystal structure destruction took place. So, it is concluded that at this stage, the maximum value of the fluctuation of the cutting force was larger. As shown in Fig. 6b, the red region took up a larger area during the later stage of simulation, which indicates the cutting force was raised markedly. Under the same simulation time, the cutting force was smaller when the cutting angle was less than 15°, which means small portion of crystal lattice was destroyed.

### Energy Analysis for the SiC Abrasive Impact Cutting Process

**Fig. 7:**
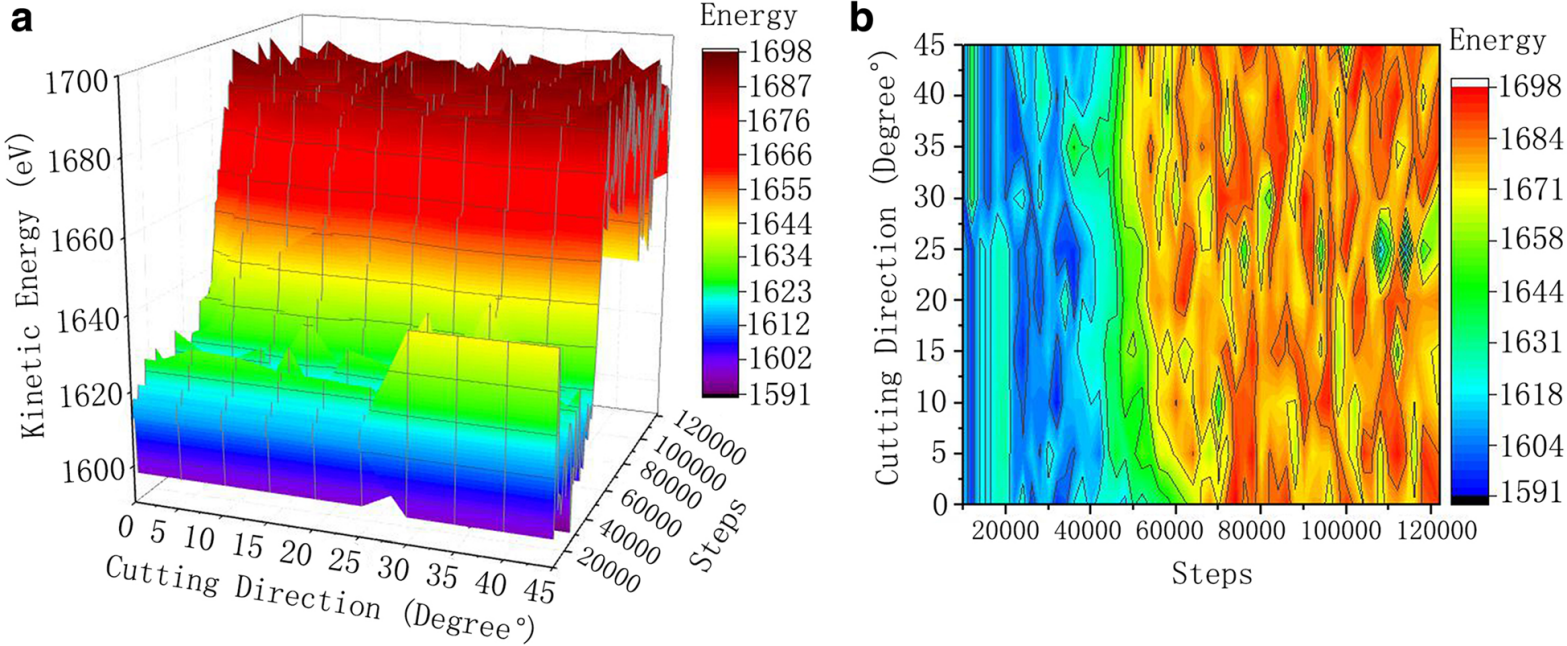
Kinetic energy curve of the workpiece atoms. Figure 7 is the kinetic energy curve of the different cutting angles during the cutting process. With the increase in the number of simulation steps, the kinetic energy of the atoms fluctuates in a low range, and then to a higher range within the fluctuations. **a** 3D curve. **b** Contour

The thermodynamics total energy is the sum of the total potential energy and total kinetic energy in a system, which reveals the effect of the work done on workpiece by abrasive grains. The influence of cutting angle on silicon carbide abrasive on the energy change of the system is shown in Fig. 7. In the process of cutting, single crystal copper workpiece materials with silicon carbide abrasive grains, the work done by abrasive grains on workpiece material acts in two ways; one part is converted into kinetic energy that increases the heat of single crystal copper atoms contact with silicon carbide particles functioning polishing, the other part is converted into potential energy which allows changes in the internal structure of the single crystal copper workpiece, the lattice deformation and the lattice energy release.

In the process of abrasive grain cutting of single crystal copper workpiece, the kinetic energy of the system is closely related to the work done of the workpiece material by SiC abrasives. By figuring up the velocity of particles in the system at every moment, the total system kinetic energy can be calculated:

1 $$ K=\sum \limits_{i=1}^N\frac{1}{2}{m}_i\left({v}_{\mathrm{ix}}^2+{v}_{\mathrm{iy}}^2+{v}_{\mathrm{iz}}^2\right) $$

In the formula, ${v}_{\mathrm{ix}}^2$, ${v}_{\mathrm{iy}}^2$, and ${v}_{\mathrm{iz}}^2$ represent the velocity component *x*, *y*, *z* direction atoms, respectively.

Kinetic curve in Fig. 7 led us to conclude that upon simulation steps increment, initially the kinetic energy of the atoms fluctuates in a low range and then into a higher range. With the movement of abrasive grains, a long-range repulsive interaction is found between the outermost copper atoms and Si, C atoms (SiC). The copper atoms in the workpiece begin to get kinetic energy and move. When the silicon carbide particles contact the single crystal of copper, the atomic temperature of the contacting region increases which rises the atomic thermal motion. When the cutting is stable, the kinetic energy of the copper atoms in the material convey and transform in a dynamic equilibrium pattern, and the kinetic energy of the atoms fluctuates in a high range.

Analysis of the kinetic energy led us to conclude that during the cutting process, when the particles begin to contact the workpiece, the atoms in the extruded region undergo lattice deformation, atomic coordinates change, and displacement changed with kinetic energy. With the abrasive particles getting into single crystal copper, the peak value of the kinetic energy of the single crystal copper workpiece appears when the workpiece enters the workpiece completely. Because the kinetic energy and potential energy of abrasive particles are merely mutually transformed. However, the energy of the whole system will not change, except for the moving particles, which enter the workpiece. In addition, the motion of silicon carbide particles forces the workpiece atoms to move simultaneously with them. The heat generated during the atomic friction is released by the kinetic energy and the strain energy release due to dislocation movement.

The conversion between heat and kinetic energy is calculated by the following formula:

2 $$ \frac{1}{2}{\sum}_i{m}_i{v}_i^2=\frac{3}{2}{nk}_B{T}_i $$

In formula, *n* is the number of atoms; vi represents the instantaneous velocity; *k*_*B*_ is the Boltzmann constant, and *T*_*i*_ is the temperature of the atoms.

The cutting force increases the atomic temperature of the area where the abrasive contact the workpiece material. On considering the thermal motion of the atom and the kinetic energy of the atoms, the kinetic energy of the copper atoms increases partially. The simulated system is set to the canonical ensemble; the overall system temperature fluctuates in a certain range. The heat produced during the cutting of abrasive particles is rapidly transferred to the constant temperature atom layer, so the overall kinetic energy of the system changes very little.

**Fig. 8:**
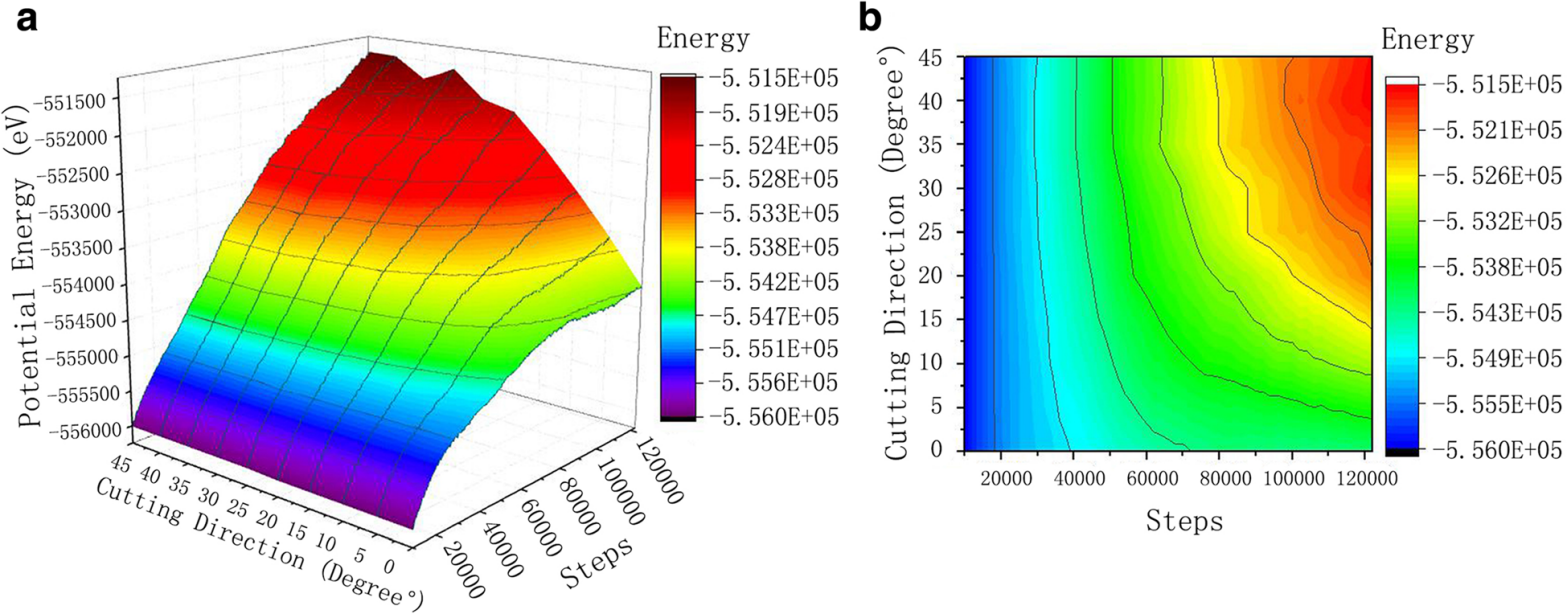
Potential energy curve of the workpiece. Figure 8 is a graph of potential energy variations at different cutting angles during the cutting process. With the increase in the number of steps for simulation, the potential between the monocrystal copper atoms exhibit workpiece tends to increase. **a** 3D curve. **b** Contour

**Fig. 9:**
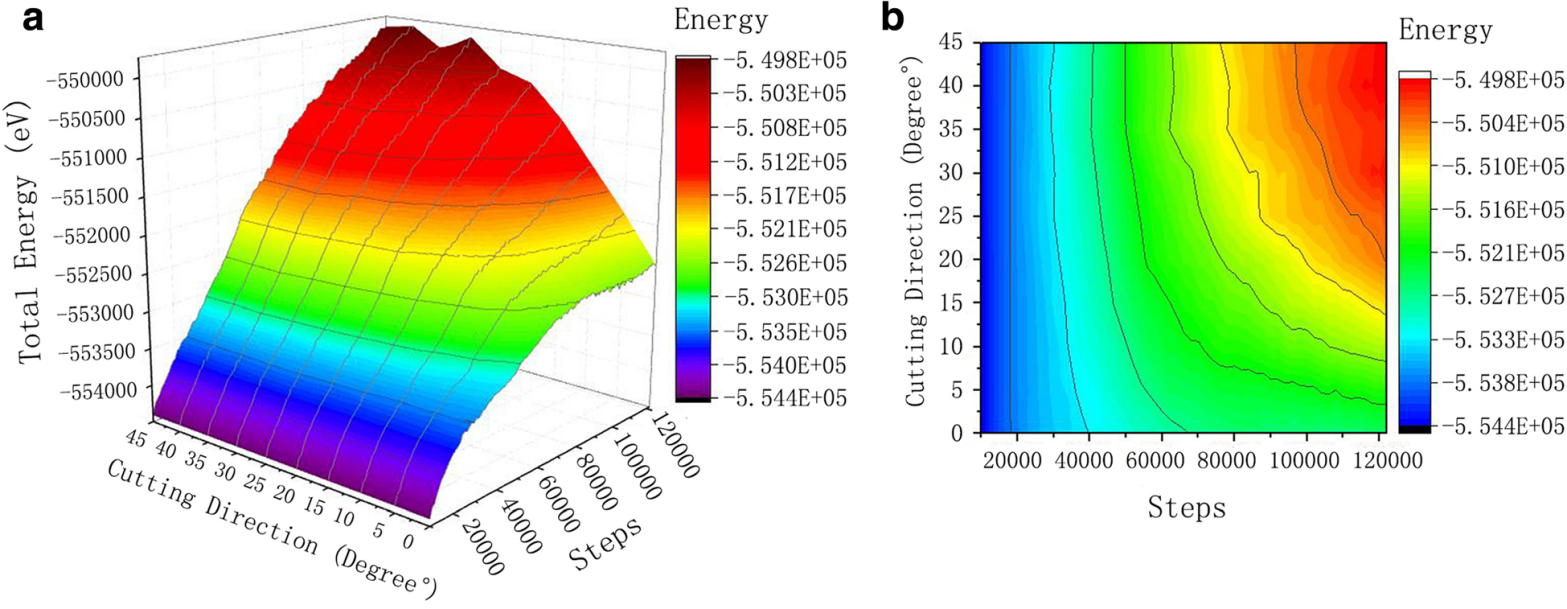
Total energy curve. Figure 9 shows the total energy curve. During the process of SiC cutting the single crystal copper workpiece, the effect of the abrasive grains on the workpiece is manifested in two forms: part of which is converted into kinetic energy, the atomic heat is increased, and local kinetic energy becomes larger when polishing the joint area of the single crystal copper workpiece and the SIC particles; the other part is converted into potential energy, the internal structure of the monocrystal copper workpiece is changed, lattice is deformed, and lattice energy is released and changed into potential energy. **a** 3D curve. **b** Contour

As can be seen from the potential energy curve of Fig. 8, upon increasing the number of simulation steps, the potential energy among the mono crystal of copper atoms in workpiece also tend to increase. When silicon carbide abrasive contact the single crystal of copper workpiece from start to fully stable cutting, the workpiece material gradually deformed and the copper atoms displace, which leads to the distortion of the crystal lattice in the crystal. This also results an elastic stress field, where the strain energy increases. When the strain energy is not enough to rearrange the material atoms, the dislocation of atoms occurs which increase the total energy of the system. Contrast to the potential curves from 0° to 45°, we can see a consistency in thermal motion of atoms at constant temperature. There is no significant relationship between the atomic kinetic energy and the cutting angle of SiC abrasive grains. The kinetic energy of the atoms is closely related to the thermal motion of atoms. However, the change of potential energy and total energy is obviously related to the cutting angle of SiC abrasive particles. The magnitude of atomic potential energy increases with increasing of cutting angle. When the cutting angle is between 0° and 20°, an obvious change in potential energy is observed. However, when the cutting angle is between 25° and 45°, potential energy remains as such, and the overall potential energy curves are from 0° to 20° cutting angle. As shown in Fig. 9, the total energy trend is similar to that of potential energy curve. The total energy values of all cutting angles are very close to one another. This is because the total energy is equal to the potential energy and kinetic energy of the system, while the kinetic energy does not change in pace with the cutting angle, during the cutting process. The change in kinetic energy of workpiece is very low with different cutting angles. Therefore, the change curve of the potential energy is similar to the curve of total energy. Comparing atomic displacement diagrams of Fig. 9, it can be found that under the same cutting condition, the cutting depth in single crystal copper workpiece is larger when the cutting angle is between 25° and 45°, which can be attributed to the increase displacement of copper atoms. Along the [001] direction of SiC particles, more atomic damaged in lattice and dislocations are generated. The strain energy produced during this time is higher, which result a high potential energy change curve and total energy change curve of the workpiece atoms.

### Analysis of Atomic Displacement in SiC Abrasive Particles Collide Cutting Process

**Fig. 10:**
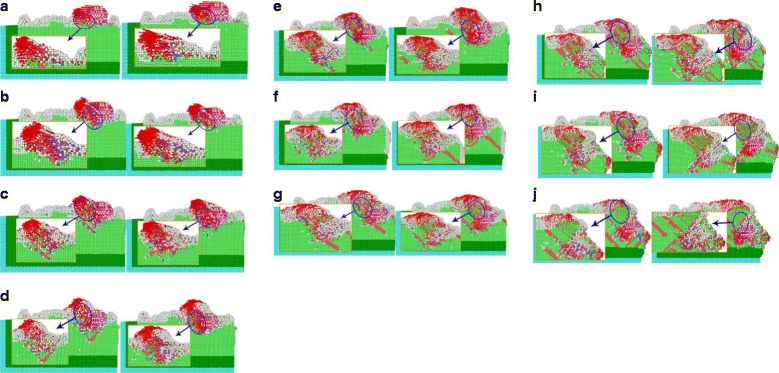
Different cutting angles cause to atomic displacements. Figure 10 describes atomic displacements of different cutting angles. When the single crystal SiC abrasive particles collide with the workpiece cutting copper in different directions, with SiC abrasive grains being cut, cutting depth increases, since the speed of the cutting direction is at 80 m/s, the [001] direction The moving speed is *v*_*z*_ = 0.8 ∗ sin *θ*, with increasing cutting depth of the cutting angle, [001] in the same cutting angle increases simulation steps, the depth of cut is large at the same time. **a** 0° displacement. **b** 5° displacements. **c** 10° displacement. **d** 15° displacement. **e** 20° displacement. **f** 25° displacement. **g** 30° displacement. **h** 35° displacement. **i** 40° displacement. **j** 45° displacement. (atomic color:  HCP structure,  BCC structure,  FCC configuration as a white amorphous structure)

In cutting process of workpiece material at various angles, the abrasive grains move along the cutting direction, forcing the copper atoms in the workpiece to move. From the analysis of the moving direction of the workpiece, the effect of the abrasive grains on the workpiece material, how the chip form, and material removal are completely clarified. According to bond angle analysis method, proposed by Ackland-Jones, different atomic lattice structure is marked by diversified colors, for observation purpose and analysis. The ZOY plane is selected to observe the cutting process of an individual SiC particle, and the atomic displacement diagram of the cutting area is enlarged, as can be seen from Fig. 10.

When the single crystal SiC abrasive particles collide with the workpiece cutting copper in different directions, the cutting depth increases along the process. The speed of the cutting direction is 80 m/s, where the component speed in [001] direction is *v*_*z*_ = 0.8^∗^ sin *θ*. At the same simulation step, cutting depth increases with increasing the cutting angle. As shown in Fig. 10, there is an atomic position shift in both of the surface and interior of the workpiece material, contacting with the abrasive grains, where atoms of different lattice types are arranged and doped with each other. Due to the movement of these abrasive particles, the copper atoms accumulated at the tip of the abrasive grains, which results no cracks during abrasive particles cutting. It can be seen that the material removal mode of the abrasive particles is plastic deformation. At the same time, due to abrasive cutting, the atoms displacement in single crystal copper increases from 15° to 45°.

The number of the workpiece atoms along the abrasive grain motion direction also increases, and the atomic displacement is perpendicular to the direction of abrasive cutting. This part of the atomic displacement is due to that of the abrasive particles, change the cutting action to the extrusion upon the workpiece atoms. At cutting angle, the abrasive particles is between 0° and 10°, while the cutting depth is smaller than those of the 15° to 45°. Abrasive particles are performing mainly the cutting function, the extrusion of the workpiece atoms is marginal, and the number of atoms is small whose displacement direction is perpendicular to the direction of the abrasive grain motion. With the repeated cutting on the workpiece surface by numerous abrasive particles, the abrasive particles having large cutting angle produce deep pits on the workpiece material during the whole cutting process, while ones following smaller cutting angles will continue cutting along the cutting mark produced by the former. Under the combined action, the workpiece material is cut to a certain depth (micro-cutting) followed by whole abrasive flow polishing.

### Dislocation Collision Analysis for SiC Abrasive Cutting

**Fig. 11:**
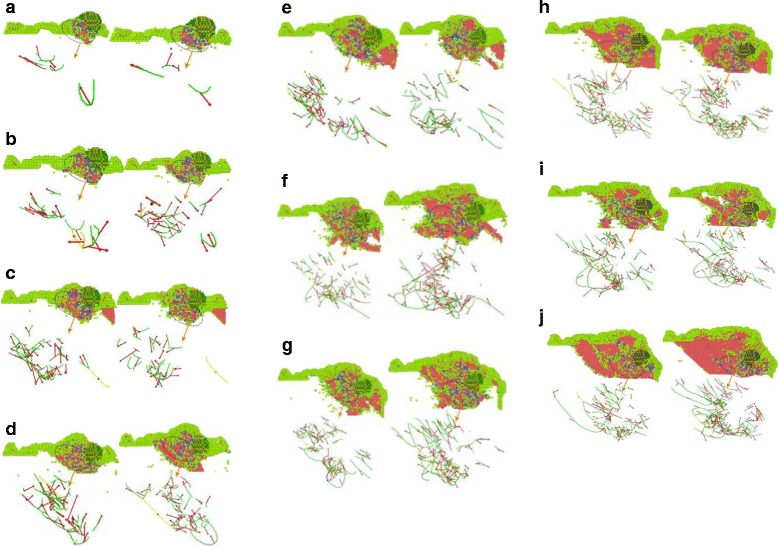
Different angles-bond angle dislocation line charts. Figure 11 represents the cutting dislocation line and the bond angle. As the repeated cuttings on the workpiece surface are done by the numerous abrasive particles during the polishing process, in the entire cutting process, the abrasive particles with larger cutting angle produce bigger pits, while the following abrasive particles with smaller cutting angles continue to polish along the cutting trace. Therefore, certain depth is done on the workpiece material and the whole micro-cutting of abrasive flow polishing is achieved. **a** Dislocation lines with 0° cutting angles. **b** Dislocation lines with 5° cutting angles. **c** Dislocation lines with 10° cutting angles. **d** Dislocation lines with 15° cutting angles. **e** Dislocation lines with 20° cutting angles. **f** Dislocation lines with 25° cutting angles. **g** Dislocation lines with 30° cutting angles. **h** Dislocation lines with 35° cutting angles. **i** Dislocation lines with 40° cutting angles. **j** Dislocation lines with 45° cutting angles. (Note: the upper part of the key angle analysis, the figure in the atomic color:  HCP  BCC  Amorphous structure  Abrasive grain; the lower part of the dislocation line diagram, the figure dislocation line color:  Stair-rod,  Shockley,  Hirth,  Perect,  Frank,  Burgers vectors)

Dislocation is a special arrangement of atoms in crystal along certain crystal surface and crystal direction, or a boundary between the slip zone and the non-slip zone on the slip surface. Dislocation can be divided into edge dislocation, spiral dislocation, and mixed dislocation, among which mixed dislocation is most common. In the process of abrasive particle cutting, the single crystal copper workpiece is plastically deformed, the atoms move, and the crystal lattice breaks and reconstructs, which results in a large number of dislocations. The analysis for dislocation and bond angles at different incidence angles is shown in Fig. 10. In the simulation model of abrasive flow simulation, two abrasive particles are used for cutting the workpiece material. For ease of analysis, the generation and change of different dislocation lines in single crystal copper material, during cutting process and the different lattice structures in the cutting part are analyzed in the view of one single SiC particle on the YOZ surface, as shown in Fig. 11.

Key angle analysis diagram of each cutting angle is shown in Fig. 11, where the sample from the interior workpiece simulation at time 60 and 70 ps, the atoms are colored individually according to different arrangement of atoms through BAD technology. To facilitate the analysis of the lattice changes in the cutting part, atoms of the FCC structure in the workpiece material are removed. By DXA techniques, different types of dislocations are marked in diverse colors: red arrow indicates the Burgers vector (Burgers vectors), green for Shockley partial dislocations (Shockley), light blue for the Frank partial dislocations (Frank), pink pole position for the pressure error (Stair-rod), and blue for the whole dislocation (Perfect).

As can be observed from the analysis of the bond angle portion in Fig. 11, a large number of dislocations and lattice deformations occurred under the impact of external cutting forces. The bond angle analysis shows that the internal HCP structure of the workpiece appears more clearly with increasing of cutting angle. On further simulations, the structure of HCP increases gradually. The silicon carbide particles continuously cut and squeeze the workpiece material at 80 m/s speed, resulting in a phase transition from the FCC structure to the HCP structure. During this phase transition, the atomic strain of the workpiece continues to increase. However, when the stress state of atoms of the workpiece has exceeded the threshold of thermodynamic phase transition, it turns to metastable state. As the strain increases, the HCP phase begins to nucleate and grow spontaneously, and the FCC lattice of copper undergoes absolute destabilization, which induces a sudden change in mechanical quantities. As the kinetic energy of the atom is directly related to the atomic temperature (formula 1), there is a saltatory augment in the kinetic energy change curve, as can be seen from Fig. 7. This is due to high strain energy, accumulated in the single crystal copper workpiece before the nucleation of HCP. The HCP structure increases the system temperature through metastable nucleation and partial stress release. Due to the abrasive cutting and extrusion, bonds of copper atoms in workpiece material break down which results in disorder. The original regular lattice structure is broken, where the partial copper atoms gradually change into disorder and form an amorphous structure. It can be seen from the bond angle analysis diagram that (Fig. 11) atoms with relatively disordered displacement tend to form amorphous structures at 60 and 70 ps, which is because of silicon carbide abrasive particles. At the same time, many atoms with the same displacement direction are transformed into HCP structures. Meanwhile, the particles shear downward and the atomic structure is rearranged due to the displacement of atoms in the original amorphous structure, results an amorphous structure of HCP. Atoms that have already been transformed into HCP structures, even before the next cut stage, change back into amorphous structure. As the cutting depth increases, the number of amorphous atoms near the abrasive particles also increases.

**Fig. 12:**
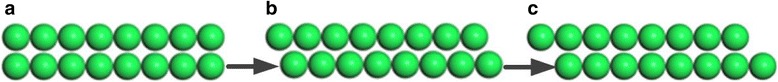
Schematic view of dislocation changes. Dislocation is a special arrangement of atoms in crystals. It is the crystal in the atomic arrangement along a certain crystal face and crystal orientation occurred in a certain wrong line, and it is the boundary of the slip surface within the area where the slip has occurred and the area with no slip has occurred. In Fig. 12**a**–**c**, the relative displacement of the rigidity of the upper atom and the underlying atom produce dislocations, the upper atom belongs to the sliding region, and the lower atoms belong to the non-slip region, they are in the slip surface of the intersection line which is called dislocation line, that is, dislocation. Figure 12 is the process of dislocation generation and development

When the FCC crystal structure undergoes cutting by abrasive particles, a shear stress generates in atoms, which results in the displacement of atoms in the lattice and the arrangement of various lattice structures. In moving the atoms of workpiece, the rigid displacements of the atoms produce dislocations, also called as dislocation lines (Fig. 11). Dislocation line can be termed as the boundary between the slip zone and the non-slip zone, on the slip surface. According to the relation between the dislocation and the PATS vector, dislocation is parallel to the PATS vector, called the screw dislocations. The vertical ones are edge dislocations, and the mixed dislocations are neither parallel nor vertical. In Fig. 11, most of the dislocation lines are neither parallel nor vertical to PATS vectors, which are typical mixed dislocations. In the cutting process of abrasive particles, the dislocation line changes, moves and grows around the abrasive particles. The closer the particle, the greater the density of the dislocation line. Dislocation lines are also very dense, when the arrangement of atoms is complicated. The abrasive particles marked as orange, produce a large amount of HCP, BCC, and amorphous structures around them, which interlace and blend with each other to form dislocations that increases the internal energy of the crystals. When atoms arrange their self just like shown in Fig. 12b, possess maximum potential energy. While for the arrangement, shown in Fig. 12a–b, the atoms situate at the lowest potential energy position. The number of dislocation lines is directly related to the magnitude of strain energy of the crystal. The total strain energy of the unit length dislocation can be measured with the given formula:

3 $$ W=\alpha {Gb}^2 $$

Where, *α* is the geometric factor (type dislocations, dislocation density) parameter related to, and generally 0.5 ~ 1.0; *G* is the shear modulus, and *b* is the slippage distance.

When the workpiece material is being cut, particles break the atomic arrangement and the lattice reconstructs, which constitutes, macroscopically, the plastic deformation of the workpiece material. In the process of plastic deformation, the dislocation was supposed to escape from the crystal and decrease the dislocation density. However, this dislocation density increases due to dislocation propagation. There are many ways of dislocation multiplication and the main one is the Frank–Reed dislocation source theory, of which the growth mechanism is shown in Fig. 11. As illustrated in Fig. 11, the density of the dislocation line increases markedly from 60 to 70 ps when the cutting simulation proceeds, change both the number and shape of dislocation lines in accordance with Frank–Reed dislocation source theory. At simulation time of 60 ps, there are many long Shockley dislocations. But after another 10 ps simulating, the long Shockley dislocation line becomes less and shorter. The original straight dislocation lines turn into bent, which is especially evident near the abrasive grains. From formula 3, the strain energy of the dislocation is proportional to *b*^2^. From the viewpoint of energy, the dislocations with the smallest *b* in the crystal should be most stable with low energy; however, dislocations with larger *b* will break down into ones. In addition, the energy of the dislocation is valued by the unit length of the dislocation line. Given the shortest line between two points is the straight, the strain energy of straight dislocation is lower than that of the bent one, means straight dislocations are more stable. Frank–Reed dislocation source theory argued that the long dislocation line becomes shorter and breaks down into smaller dislocations, thus decreasing the strain energy of the crystal.

### Analysis for Friction Coefficient Between Workpiece Surface and Abrasive Particles

To quantitatively disclose mechanical properties and surface effect of SiC abrasive cutting single crystal copper material, the tangential force ([010] direction) and normal force ([001]direction) on the cutting surface are further analyzed. The friction coefficient can be defined as the ratio of the tangential force to the normal force, with the formula below:

4 $$ f=\frac{F_y}{F_z} $$

**Fig. 13:**
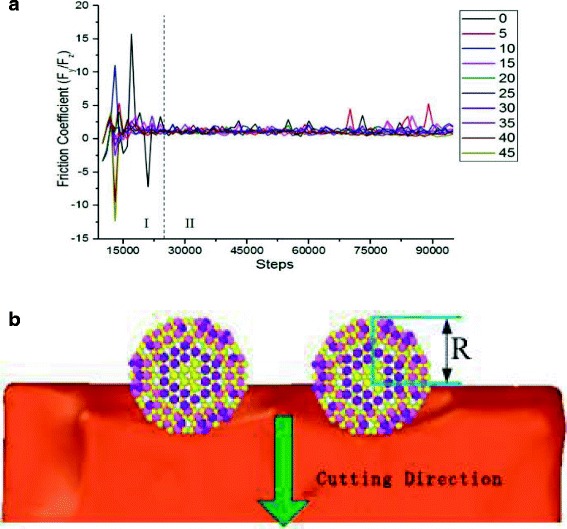
Friction coefficients of different cutting angles. Figure 13 is the changes of the frictional coefficients during SIC particles cutting single crystal copper from different angles. The change in cutting angle did not cause significant changes in friction coefficient. It described two kinds of coefficient of friction in contact with the relevant atoms in the cutting process, regardless of the cutting mode. **a** Friction coefficient variation curve with cutting angle. **b** Instantaneous structure when SiC abrasive grains move 7.5 Å

Figure 13 shows the variation of friction coefficient along different cutting angles during abrasive cutting. The change of friction coefficient can be divided into two periods. Period I, when the cutting distance is less than half of the size of the abrasive particle, the friction coefficient fluctuates violently in certain range due to surface effect of the material; while in period II, all the friction coefficients fluctuate in very small range, and the friction coefficients remains steady state at different angles. However, at cutting angle of 5°, there is a small amount of abnormal fluctuation at the end of simulation. Moreover, in period I, abrasive particles contact the workpiece atoms to the abrasive grains enter into the workpiece of half abrasive diameter (shown in Fig. 13b), the cutting movement distance reaches 7.5 Å. In Fig. 6, the cutting force change curve shows that the tangential force and normal force are in an oscillating phase during period I. Since the friction coefficient can be characterized as the adhesion among atoms in the contact surface and is related to the two atoms contacting with each other, regardless of the cutting mode, which can interpret the phenomenon that change of cutting angle does not cause significant change of friction coefficient, as shown in Fig. 13a.

## Conclusions

The micro cutting simulation of single crystal copper by SiC abrasive particles is achieved during abrasive flow polishing. Comprehensive analysis of the SiC abrasive cutting reveals that when SiC abrasive particles cut at angle from 0° to 15°, cutting forces in along *Y* and *Z* direction are relatively small and tend to stay steady. A larger cutting angle results more dislocations, which brings about larger grooves damaging of the workpiece material. In the process of cutting, it is necessary to avoid more dislocations, which has an immediate adverse effect on the performance of all aspects of crystal material. Therefore, a cutting angle of 0° is better than other angles. Considering the amount of dislocation produced, or the change of cutting force and energy in the cutting process; cutting with small or medium angles is not only beneficial for improving surface quality but reduce the internal defects.
